# Modelling Stress-Dependent Magnetic Permeability Using Two-Domain Approach with an Effective Anisotropic Wall Energy in Grain-Oriented Electrical Steel

**DOI:** 10.3390/ma19020274

**Published:** 2026-01-09

**Authors:** Tadeusz Szumiata, Roman Szewczyk, Paweł Rękas, Michał Nowicki

**Affiliations:** 1 Faculty of Mechanical Engineering, Department of Physics, Kazimierz Pulaski Radom University, Stasieckiego 54, 26-600 Radom, Poland; t.szumiata@urad.edu.pl; 2 Faculty of Mechatronics, Warsaw University of Technology, sw. Andrzeja Boboli 8, 02-525 Warsaw, Poland; pawel.rekas.dokt@pw.edu.pl (P.R.); michal.nowicki@pw.edu.pl (M.N.)

**Keywords:** grain-oriented electrical steel, magnetoelastic effect, domain walls motion, energy minimization, effective anisotropic wall energy

## Abstract

The magnetoelastic effect in grain-oriented electrical steels arises from interactions between magnetocrystalline anisotropy, domain wall confinement, and applied mechanical stress. This presents a comprehensive model based on the minimization of total magnetic energy in a two-domain system separated by a 180° Bloch wall. The model uniquely permits independent variation in the magnetization angle and external field direction, allowing accurate representation of energy competition among magnetostatic coupling, inter-domain interactions, and multi-component anisotropic confinement. The effective anisotropic wall energy incorporates isotropic, uniaxial, and six-fold crystallographic anisotropies modified by stress-induced terms. The Bloch wall position and the actual direction of magnetization are the variables that minimize the energy. Transformation to dimensionless variables enables efficient parameter identification via tri-division search. Experimental validation on M120-27s grain-oriented steel demonstrates that the model quantitatively reproduces stress-dependent 2D permeability tensors across arbitrary cutting orientations with very good quality, confirmed by determination coefficient R-squared exceeding 98%, which verifies the physical validity of the proposed model. This satisfactory agreement, together with the concept of anisotropic domain wall effective energy, represents a genuine novelty in the analysis of low-field magnetic permeability in grain-oriented electrical steels.

## 1. Introduction

Grain-oriented electrical steels (GOES) are the key materials for high-efficiency transformers and rotating electrical machines [1,2]. Due to their high permeability along the rolling direction, these materials enable low power losses and compact designs. As a result, yearly industrial consumption of grain-oriented electrical steels exceeds 3.1 million metric tons [3]. However, industrial applications subject electrical steels to complex magnetomechanical loading conditions that deviate significantly from idealized uniaxial magnetization under stress-free conditions. Manufacturing and exploitation processes, including cutting [4], punching [5], clamping, and mechanical assembly to maintain lamination stack integrity [6], introduce both residual and elastic-type mechanical stresses, significantly changing the magnetic characteristics of grain-oriented electrical steels [7,8].

As a result, magnetoelastic characteristics of grain-oriented electrical steels have great technical importance. However, understanding the physical mechanism behind the magnetoelastic effect in grain-oriented electrical steels requires a quantitative model of interaction between the magnetoelastic energy and magnetocrystalline energy in strongly anisotropic magnetic materials. It is worth noting that understanding this interaction on a micromagnetic scale is not possible using classical physics and requires consideration of quantum phenomena [9,10].

On the other hand, the magnetoelastic effect has been widely described on the macroscopic scale using the Jiles–Atherton model [11,12,13], with extensions introduced by Sablik [14]. There is also a group of phenomenological models that describe magnetoelastic effects based on polynomial interpolation [15] or neural networks [16].

However, such approaches to modelling the magnetoelastic effects in grain-oriented electrical steels create a limited possibility of a quantitative description of the influence of mechanical stresses on the 2D relative permeability tensor. Such a description is especially important for finite element modelling of the magnetic properties of electrical transformers subjected to mechanical stresses [17,18], which has great technical importance. A previously proposed model based on a planar system of two domains with a movable domain wall between them [19] partially addressed this problem for the magnetizing field vector parallel to the flux density.

The new proposed model better reproduces the physical background behind the stress-dependent effective anisotropic energy of domain walls in grain-oriented electrical steels. It creates the possibility of more accurately modelling the influence of mechanical stresses on the functional characteristics of power transformers with the cores made of grain-oriented electrical steels. Compared to the previous approach [20], delivering only a qualitative reproduction of the directional dependence of magnetic permeability, the present work provides a satisfactory quantitative agreement with experimental data.

## 2. Conception of the Model

**Figure 1:**
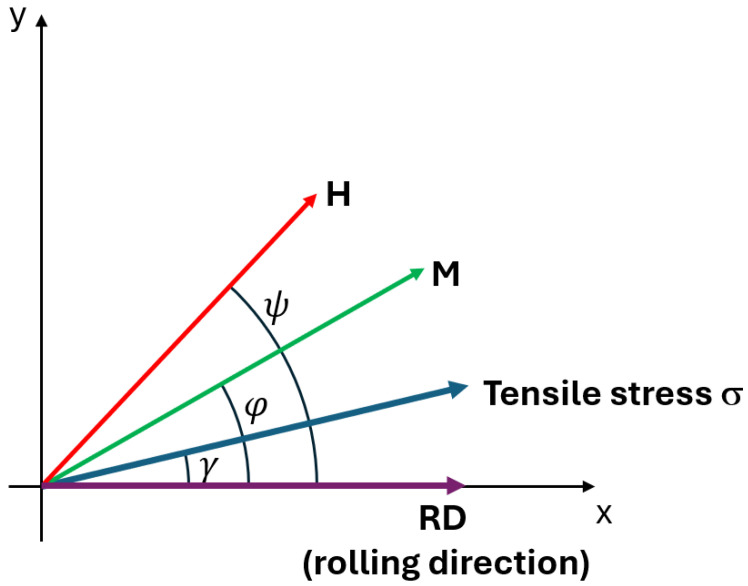
The angles γ, ψ, and φ for angles against the rolling direction (in X axis direction): tensile stresses *σ*, magnetization *M*, and magnetizing field *H*, respectively.

To fulfil the need for a quantitative description of the 2D magnetization process in grain-oriented electrical steels under mechanical stresses, an approach is presented that directly describes the translation of a 180° domain wall in a two-domain system through total-energy minimization. The proposed model is based on a planar system consisting of two domains with a movable domain wall separating them, as described in detail in [19,20]. However, in the present, more general case, the actual in-plane magnetization vector angle ψ is not assumed to be the same as the in-plane external magnetic field angle φ, as shown in Figure 1.

The magnetostatic energy density of this system in the external magnetic field of induction $B_{0}$ is given by the following formula:

(1) $${E_{B}}_{0}\left(x,\psi \right)=-2B_{0}M_{s}\frac{x}{d}\cos\left(\psi -\varphi \right),$$

where $M_{s}$ is saturation magnetization, d is the width of the two-domain system, and x is the position of the domain wall (±d/2).

In general, both subdomains in a two-domain system can interact with each other, and, according to [19,20], the energy of such magnetostatic coupling can be estimated as follows:

(2) $$E_{\mathrm{ms}}\left(x\right)=-\eta \mu_{0}{M_{s}}^{2}\left[\frac{1}{4}-{\left(\frac{x}{d}\right)}^{2}\right],$$

where $\mu_{0}$ is the magnetic permeability of vacuum, and η is the dimensionless coefficient dependent on the size and shape of the subdomains.

The key mechanism responsible for the magnetization in weak external magnetic fields is the confinement of domain walls. When assuming a parabolic form of the anisotropic confinement well, the density of effective anisotropic wall energy can be expressed as follows [19,20]:

(3) $$E_{\mathrm{an}}\left(x,\psi \right)=\frac{1}{2}P\left(\psi \right){\left(\frac{x}{d}\right)}^{2},$$

where $P\left(\psi \right)$ is an effective anisotropic stiffness coefficient which depends on the magnetization angle ψ, measured with respect to the rolling direction. It should be indicated that in classical approaches to domain wall dynamics, the decomposition of magnetization into reversible and irreversible components is necessary due to the path-dependent nature of domain wall motion across energy barriers. The present model specifically addresses the reversible, low-field regime where domain walls remain confined within parabolic potential wells and do not overcome such barriers. In such a regime, the equilibrium position depends solely on the current energy balance, not on the magnetization history. As a result, no decomposition into reversible and irreversible parts is required.

In the present approach, this coefficient considers both isotropic contribution ($P_{iso}$), uniaxial and six-fold anisotropies resulting from the rolling process (coefficients ${P_{0},Q}_{0}$), and similar terms with coefficients ${P_{s},Q}_{s}$ corresponding to external stress σ applied at γ angle with respect to rolling direction [19]:

(4) $$P\left(\psi \right)={P_{iso}+P}_{0}{cos}^{2}\left(\psi \right)+Q_{0}{sin}^{6}\left(\psi \right)+P_{s}\cos^{2}\left(\psi -\gamma \right)+Q_{s}\sin^{6}\left(\psi -\gamma \right).$$

It should be highlighted that the angular-dependent terms in the effective anisotropic stiffness coefficient (Equation (4)) are consistent with the general framework of the spherical harmonic decomposition of magnetic anisotropy energy, as developed by Néel [21] and systematically treated by du Tremolet de Lacheisserie [22]. In the general 3D formulation, the magnetoelastic and magnetocrystalline anisotropy energies are expressed as expansions in spherical harmonics. For thin electrical steel sheets where the magnetization lies predominantly in the sheet plane, this 3D formulation reduces to a planar projection, yielding the ${cos}^{2}\left(\psi \right)$ and ${sin}^{6}\left(\psi \right)$ angular dependencies utilized in the present model.

It should be highlighted that *P*s and *Q*s are obviously *σ* functions. However, *P*_0_ and *Q*_0_ can also depend on *σ* because stress *σ* not only introduces anisotropy in the *γ* direction but also influences all other parameters—including the rolling anisotropy state, and even *P*_iso_, the isotropic part of the effective anisotropic wall energy. The definite form (4) of the anisotropic wall effective energy coefficient *P*(*ψ*) was chosen to obtain possible good accordance of predicted anisotropy of magnetic permeability with experimental outcomes and to avoid an excess of the number of fitting parameters. The isotropic term represents orientation-independent confinement arising from randomly distributed defects. The angular terms reflect the complex nature of confinement-centre deformation under applied stress. A specific form of the confinement anisotropy was selected to ensure the best agreement between simulation results and experimental data, while maintaining the simplicity of the model. The functional forms in Equation (4) are based on symmetry considerations.

The ${cos}^{2}\left(\psi \right)$ terms represent axial anisotropy: this is the lowest-order even function consistent with a preferred axis induced by rolling or applied stress and appears in standard treatments of uniaxial magnetic anisotropy. The$\;{sin}^{6}\left(\psi \right)$ terms are phenomenological higher-order contributions capturing angular variation not described by axial terms alone. This form vanishes along the rolling direction where axial confinement dominates and provides the higher-order angular dependence needed to match experimental observations. Alternative forms using ${sin}^{4}()$ were tested but did not improve fitting quality, while odd-power terms are forbidden by the symmetry requirement. The specific functional form represents an optimized balance between model complexity and predictive accuracy.

Finally, the magnetic energy of a two-domain system is a sum of above-mentioned terms,

(5) $$E\left(x,\psi ,\sigma \right)={E_{B}}_{0}\left(x,\psi \right)+E_{\mathrm{ms}}\left(x\right)+E_{\mathrm{an}}\left(x,\psi \right),$$

and it is a function of two degrees of freedom, i.e., x and ψ. Mechanical stresses are considered by the σ-dependent and orientation-sensitive anisotropic confinement in Equations (3)–(5) without including a separate elastic or magnetoelastic energy term.

It should be noted that mechanical stresses are incorporated in the present model through stress-dependent modification of the anisotropic confinement parameters rather than through an explicit magnetoelastic energy term, as employed in Jiles–Atherton–Sablik approaches [14]. This phenomenological treatment is justified by the observation that in the low-field regime, stress primarily affects magnetic behaviour by modifying the confinement environment around defects rather than by altering the equilibrium orientation of magnetization within domains through magnetoelastic effects. The applied stress changes the local strain field around pinning sites, modifying both the depth and anisotropy of anisotropic confinement wells. This mechanism is captured by the stress-dependent coefficients $P_{s}(\sigma )$ and $Q_{s}(\sigma )$ in Equation (4), which are identified from experimental permeability measurements. While this approach does not require explicit knowledge of magnetostriction coefficients, it enables accurate computation of stress-dependent permeability tensors, which represent the primary quantity required for finite element modelling of transformer cores under mechanical stresses.

In the equilibrium state in static (or quasistatic) circumstances, the magnetic energy reaches a minimum. Due to the mathematical form of the E(x,ψ) function, such a minimum cannot be determined analytically. In order to facilitate numerical identification of the parameters based on the optimization process, a transformation into dimensionless parameters and variables was performed. In the first step, the dimensionless domain wall position was introduced:

(6) $$\xi =\frac{x}{d},$$

as well as the dimensionless strength of the external magnetic field:

(7) $$h_{0}=\frac{H_{0}}{M_{s}}=\frac{B_{0}}{{\mu_{0}M}_{s}}.$$

Then, the magnetic energy $E\left(x,\psi ,\sigma \right)$ was normalized by $4\mu_{0}M_{s}^{2}$ and transformed to the dimensionless form $f\left(\xi ,\psi ,\sigma \right)$,

(8) $$f\left(\xi ,\psi ,\sigma \right)=-\frac{1}{2}h_{0}\xi \cos\left(\psi -\varphi \right)-\frac{1}{4}\eta \left(\frac{1}{4}-\xi^{2}\right)+\frac{1}{2}\xi^{2}[p_{iso}\left(\sigma \right)+\;+p_{0}\left(\sigma \right)\cos^{2}\left(\psi \right)+q_{0}\left(\sigma \right)\sin^{6}\left(\psi \right)+p_{s}\left(\sigma \right)\cos^{2}\left(\psi -\gamma \right)+q_{s}\left(\sigma \right)\sin^{6}\left(\psi -\gamma \right)]$$

containing the set of dimensionless parameters ($p_{iso},p_{0},q_{0},p_{s},q_{s}$), which depends on the external stress σ. The normalized, total magnetization of two-domain system can be expressed as follows:

(9) $$m(\xi_{opt})=\frac{M}{M_{s}}=2\xi_{opt},$$

where $\xi_{opt}$ is the optimal dimensionless position of the domain wall corresponding to the minimum of the normalized energy f(x,ψ) function. Hence, considering the component of magnetization parallel to the magnetizing field, one can write the following expression for the relative magnetic permeability $\mu_{r}$ of the system,

(10) $$\mu_{r}\left(\varphi \right)=1+\frac{m\left(\xi_{opt}\right)}{h_{0}}cos\left(\psi_{opt}-\varphi \right),$$

evaluated as $h_{0}\to 0^{+}$, where $\psi_{opt}$ is a magnetization direction in the minimum of the energy. It should be highlighted that the $h_{0}\to 0^{+}$ is evaluated within the model via energy minimization, representing the initial permeability. Experimentally, measurements performed at 750 A/m and at lower values confirmed that in this range the domain wall motion remains reversible, and permeability values coincide with the extrapolated limit $h_{0}\to 0^{+}$ validating this choice as representative of the initial permeability.

In reality, the value of relative magnetic permeability $\mu_{r}$ (obtained owing to the energy minimization) is a function of magnetic field direction φ and depends on all parameters of the model, i.e., ${p_{iso},p}_{0},q_{0},p_{s},q_{s}$, and η, the values of which can be found by fitting $\mu_{r}(\varphi )$ theoretical curves to the experimental data. In further considerations, the η parameter was taken as zero because the magnetostatic interaction energy of two subdomains (2) has a similar quadratic form as the effective anisotropic wall energy (3); thus, it contributes in the same way as the isotropic part of (4), and it would not be distinguishable for the fitting procedure. The $\mu_{r}$ (via all specified parameters of the model related to the effective anisotropic wall energy) varies along with the applied external stress σ.

It should be highlighted that the model employs five dimensionless parameters for each stress condition: $p_{iso}$ representing isotropic confinement, *p*_0_ and *q*_0_ representing rolling-induced uniaxial and higher-order anisotropies, and *p_s_* and *q_s_* representing stress-induced uniaxial and higher-order anisotropies. The inter-domain magnetostatic coupling parameter eta was set to zero because its quadratic dependence on ξ renders it mathematically indistinguishable from the isotropic confinement term during parameter identification. This five-parameter formulation is comparable in complexity to established approaches such as the Jiles–Atherton model, while providing explicit angular dependence of permeability required for 2D characterization.

The main mechanism governing low-field magnetic permeability in ferromagnetic materials is the movement of domain walls within effective anisotropic wall energy wells, in contrast to the high-field, hysteretic case, where magnetocrystalline anisotropy plays a crucial role. In the low-field regime, strong magnetocrystalline anisotropy can stabilize part of the magnetization along a single direction; however, in rolled steel sheets, a distribution of anisotropy orientations arises as a result of intense plastic deformation. The developed model of low-field anisotropic magnetic permeability does nor consider the magnetocrystalline anisotropy. One of the justifications for this is the fact that in the unstrained polycrystalline cubic system, such anisotropy averages to zero. However, after rolling, strong uniaxial anisotropy arises, and in principle it should prevent the coherent rotation of magnetization vectors in the two-domain effective approach. In such a picture, it should be impossible to magnetize the sample perpendicular to the rolling direction, but from the experiment, it is clear that even in this case, the magnetic permeability is non-zero and takes considerable values for grain-oriented steel. This presumably suggests the existence of the angular distribution of rolling anisotropy caused by strong, plastic deformation during this technological process. Thus, within the effective picture, a part of the magnetization vectors in the two-domain system is allowed to rotate and, in general, follow the magnetizing field direction. The deviations of magnetization from this direction (considered in the present model) originate from the anisotropy of the domain wall effective anisotropic energy, which is affected by both rolling and external stress. The two-domain model does not consider the evolution of real domain structure along with magnetizing, sample cutting, or straining; however, it reflects well the anisotropy of measured magnetic permeability.

In a real multidomain system, such as grain-oriented rolled transformer sheets, the magnetocrystalline anisotropy energy prevents coherent rotation of local magnetization vectors in low magnetic fields. However, experimental measurements reveal considerable magnetic permeability even in the direction perpendicular to the rolling direction. This observation suggests that, due to the angular distribution of magnetocrystalline anisotropy axes arising from imperfect texture, a fraction of the magnetization can follow the magnetizing field direction. The proposed two-domain system represents precisely this fraction of the magnetization within a simplified, effective description.

It should be stressed that the effective anisotropic wall energy introduced in Equation (3), combined with the angular-dependent stiffness coefficient in Equation (4), represents the combined contribution of magnetocrystalline anisotropy (arising from the imperfect Goss texture) and magnetoelastic anisotropy (arising from applied mechanical stress). In the broader magnetism literature, the term “pinning energy” typically refers to energy barriers associated with domain wall interactions with crystallographic defects, which govern coercivity and the Rayleigh regime. To avoid terminological confusion, the present model utilizes the term “effective anisotropic confinement energy” to describe the quantity in Equation (3). Moreover, the model parameters could equivalently be fitted to the anhysteretic magnetization curve, as the present approach characterizes the equilibrium response of the material.

It is important to note that the present model specifically addresses the initial permeability regime where domain wall motion remains reversible within the anisotropic confinement wells. The parabolic form of the anisotropic confinement (Equation (3)) is valid precisely in this low-field limit, where domain walls undergo small displacements without overcoming energy barriers that would lead to irreversible jumps and hysteretic behaviour. Consequently, validation through hysteresis curve reproduction is not applicable to this model; hysteresis characterizes the regime of irreversible domain wall motion at higher field amplitudes. As a result, validation is performed through comparison with directional permeability measurements.

In addition, the present model addresses the Villari effect, describing how applied mechanical stress modifies magnetic permeability, rather than magnetostriction, which quantifies magnetization-induced strain. In the low-field regime where magnetization changes occur through 180° domain wall motion, net magnetostriction is minimal because antiparallel domain states exhibit identical magnetostrictive strain. In principle, the magnetizing field can generate the strain in the material due to the direct magnetostriction effect. However, even in the saturation case, the magnetostrictive strain is at least 2–3 orders-of-magnitude lower than the strain caused by rolling and the external stress applied. That is why, in our model, the magnetostriction is not considered to reduce the number of significant parameters.

It should be emphasized that the present work introduces several fundamental advances beyond the framework established in [19,20]. First, the model now permits independent variation in the magnetization angle psi and the external field angle phi, eliminating a key simplification that limited the physical accuracy of previous approaches. Second, the effective anisotropic wall energy formulation has been extended to include higher-order anisotropy terms (Q_0_ and Q_s_ with sin^6^() angular dependence), which are essential for capturing the complete angular dependence observed experimentally. Third, while [19,20] achieved only qualitative agreement with experimental trends, the present model demonstrates quantitative predictive capability with a determination coefficient R^2^ exceeding 98%. These advances represent a substantial extension of the theoretical framework

## 3. Method of Experimental Measurements

The measurements were carried out on nine strips of grain-oriented M120-27s electrical steel sheets produced by STALPRODUKT S.A. (Bochnia, Poland). The thickness of the steel sheet was 0.27 mm. The test samples were cut under the following angles γ to the rolling direction using the water jet technique: 0°, 11°, 23°, 34°, 45°, 54°, 56°, 68°, and 90°.

**Figure 2:**
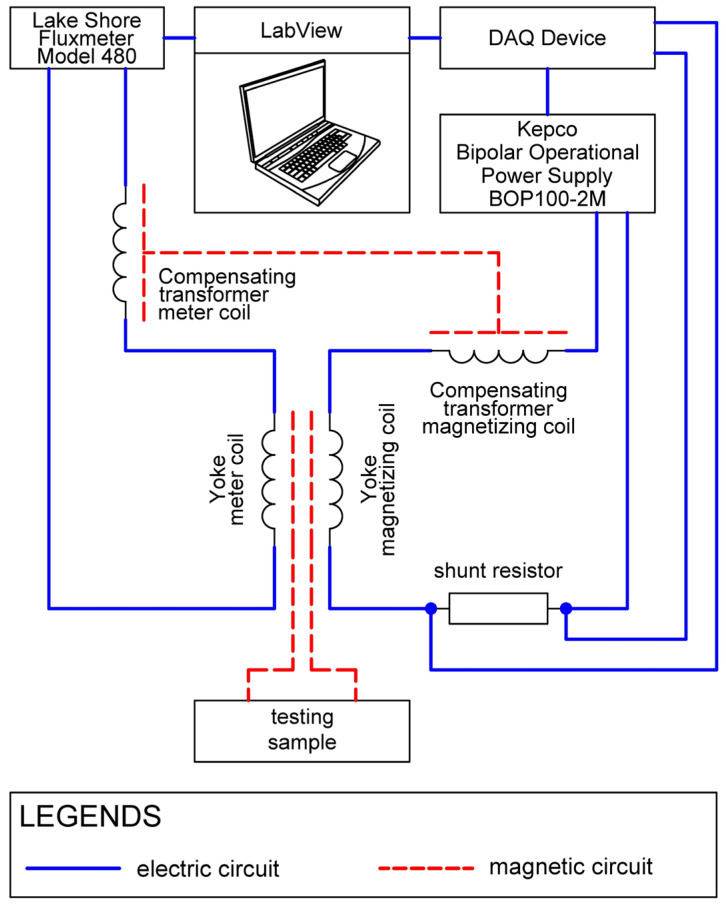
Schematic block diagram of the measuring system, including its electrical and magnetic circuits.

A schematic block diagram of the measuring system is presented in Figure 2. In the developed system for characterizing electrical steel sheets, a yoke-type probe is used [23]. A PC equipped with LabVIEW and data acquisition and control card DAQ controls a bipolar Kepco BOP100-2M high power voltage-to-current converter (Kepco, Inc., Flushing, NY, USA) to drive current through the yoke’s magnetizing coil, while a precision, low-inductance shunt resistor enables a wide range of current measurements. A Lake Shore 480 fluxmeter (Lake Shore Cryotronics, Inc., Westerville, OH, USA) integrates the voltage from a search coil to obtain magnetic flux acquired by DAQ to LabVIEW for processing.

To isolate the sample’s contribution, a small compensating transformer is excited by the same drive current and fitted with its own search coil. The two search-coil outputs are wired to subtract, which leads to compensating for the background from the yoke and fixture.

**Figure 3:**
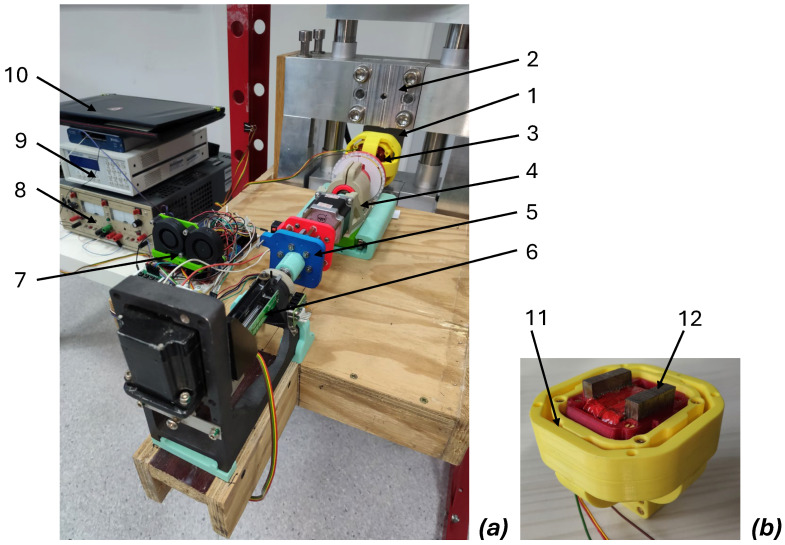
The mechanical part of the developed system: (**a**) general view of the system, (**b**) yoke-based probe with Cardan joint gimbal. 1—tested sample made of electrical steel, 2—setup to apply the axial mechanical stresses to the sample, 3—gimbal, 4—rotation driver, 5—springs, 6—linear driver for pressure, 7—DAQ acquisition card with power supply, 8—KEPCO BOP100-2M high-power voltage-to-current converter, 9—Lake Shore 480 fluxmeter, 10—PC, 11—Cardan joint in the gimbal, 12—yoke probe.

The mechanical part [23] of the developed system is presented in Figure 3. The key element of the measuring system is the Cardan joint gimbal (3, 11), enabling efficient rotation of the yoke-based probe (12) to measure 2D relative permeability of the tested electrical steel sheet. The system consists of the rotation driver (4), the set of pressure springs (5), and the linear motor (6). To enable application of compressive and tensile forces, the test samples made of electrical steel sheets (1) are mounted in a specialized mechanical force reverser (2). The entire mechanical system is made of non-magnetic materials (aluminum and austenitic steel) to avoid magnetic bias. However, in the case of electrical steel sheets, only the tensile stresses were created to avoid buckling of the test sample. Moreover, to ensure the uniform distribution of the mechanical stresses in the tested area of the sample, an optimized mechanical shape of the sample was proposed [23].

The samples were cut from the sheet of M120-27s electrical steel using the water jet technique. The direction of the samples was carefully localized against the steel sheet rolling direction, with an uncertainty of less than 1°. Measurements were carried out for the sine-wave driving current generating the amplitude of the magnetizing field $H_{m}$ equal to 750 A/m. Detailed analyses of the accuracy of the relative magnetic permeability measurements are presented in [23]. Analyses indicate that the overall uncertainty of measurements does not exceed 2%.

The 2% uncertainty was taken into account during the optimization process by evaluating the sensitivity of fitted parameters to perturbations of the measured permeability values. The resulting variation of identified parameters was significantly lower than the statistical dispersion caused by the naturally existing discrepancies between experiment and simplified theoretical model.

In addition, the tested stress range up to 94.3 MPa corresponds to typical mechanical stresses encountered during transformer manufacturing: cutting and punching operations induce residual stresses up to 100 MPa near edges, while clamping and assembly stresses typically reach 50–80 MPa. Additional stresses of similar magnitude can arise during operation due to thermal expansion and electromagnetic forces, confirming the industrial relevance of the investigated stress range.

## 4. Identification of Model Parameters and Results of Modelling

Parameters of the model were identified during the optimization process. The target function T for minimization was defined as follows:

(11) $$T=\sum_{i=1}^{N}{\left(\mu_{meas}\left(\varphi_{i}\right)-\mu_{model}\left(\varphi_{i}\right)\right)}^{2},$$

where $\mu_{meas}\left(\varphi_{i}\right)$ and $\mu_{model}\left(\varphi_{i}\right)$ are the results of measurements and modelling, respectively, whereas $\varphi_{i}$ is the angle between the main axis and magnetization field H direction and N is the total number of samples. The minimization procedure was realized numerically with the iterative tri-division method (ternary search) in the 2D space of variables [24]. The tri-division method is well-suited for optimization over two variables. Its simplicity, combined with efficiency, is particularly advantageous when energy minimization constitutes only a part of the time-consuming, primary numerical optimization of *χ*^2^ within the fitting procedure. An effective and stable algorithm for finding global minima (without utilizing derivatives) was written in Visual Basic for Applications for MS Excel.

**Figure 4:**
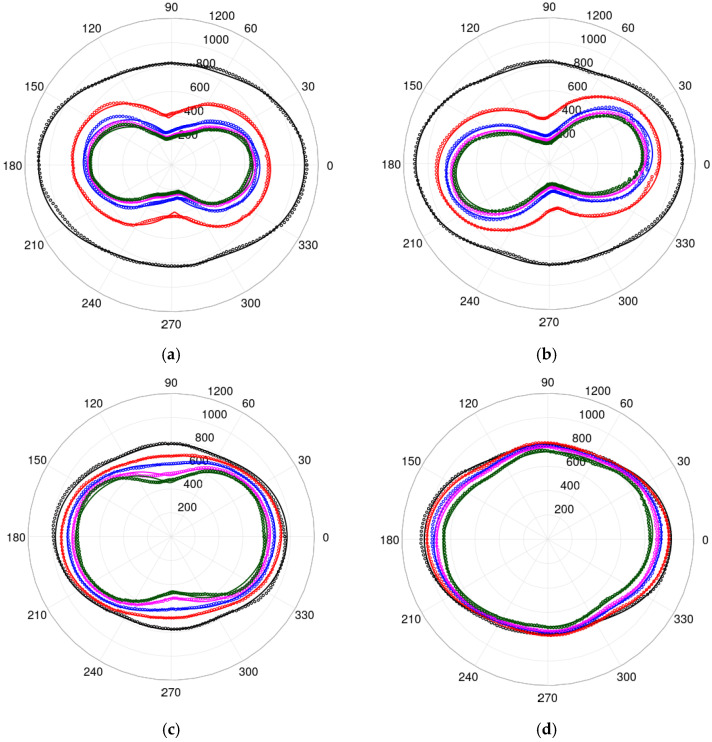

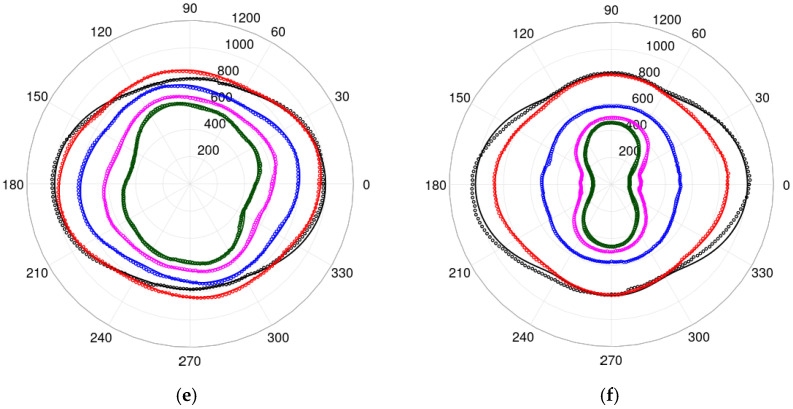
Value of 2D relative magnetic permeability of electrical steel sheet for γ: (**a**) 0°, (**b**) 23°, (**c**) 45°, (**d**) 54°, (**e**) 68°, (**f**) 90°. Tensile stresses σ are applied at an angle *γ* to the rolling direction RD (located on the *X*-axis). Tensile stresses σ are equal, 0 MPa (black), 18.9 MPa (red), 44.0 MPa (blue), 69.1 MPa (magenta), 94.3 MPa (green), applied in the direction of γ=0. Circles: measurements for different values of φ. φ=0 is the *X*-axis direction; solid lines: results of modelling.

Figure 4 presents a comparison between the modelling results and the measurement results. It can be observed that the proposed model reproduces the experimental data very well for a wide range of tensile stresses σ and for the complete range of angles γ describing the direction of the sample strip cut.

**Figure 5:**
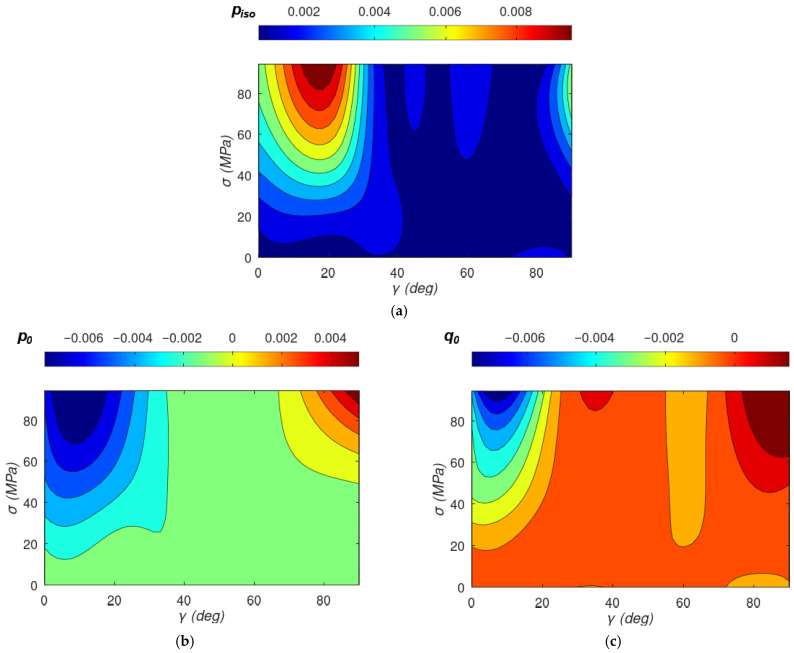

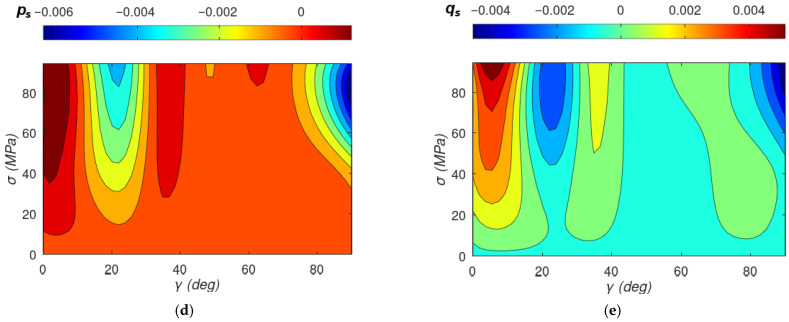
The results of the process of identification of the model parameters as a function of mechanical stresses σ applied under the direction of γ to the rolling direction: (**a**) *p_iso_*(σ,γ), (**b**) *p*_0_(σ,γ), (**c**) *q*_0_(σ,γ), (**d**) *p_s_*(σ,γ), (**e**) *q_s_*(σ,γ).

The results of the optimization-based identification of parameters piso(σ,γ), p_0_(σ,γ), *q*_0_(σ,γ), *p_s_*(σ,γ), and *q_s_*(σ,γ) on the experimental results are presented in Figure 5. For mechanical stresses, σ equals 0; the stress-induced confinement parameters *p_s_*(σ,γ) and *q_s_*(σ,γ) are equal to 0, so γ does not affect the magnetic energy of the magnetic material. However, for significant values of stresses σ, total magnetic energy dependence on γ appears through the components $p_{s}\left(\sigma \right)\cos^{2}\left(\psi -\gamma \right)$ and $q_{s}\left(\sigma \right)\sin^{6}\left(\psi -\gamma \right)$. In addition, an implicit modification of the effective anisotropic wall energy and electrical steel rolling-induced anisotropies also changes p_0_(σ,γ) and q_0_(σ,γ) as well as piso(σ,γ). As a result, the effective γ dependence observed in the fitted parameters can be richer than the explicit stress term alone due to the fact that it is influenced by both the direct magnetoelastic contribution aligned with γ and the mechanical stress-induced renormalization of *p_iso_*(σ,γ), *p*_0_(σ,γ), and *q*_0_(σ,γ) parameters.

Interpolation of discrete values of *σ*, *γ*, and parameters on colour maps is achieved by constructing a triangular mesh from the scattered points and estimating values inside each triangle through linear interpolation.

The quality of the model’s fit to the experimental data is very good. This quality is confirmed by the *R*^2^ determination coefficient, which generally exceeds 98%. A worse quality of the model, with R^2^ exceeding 90%, was observed for gamma values of about 68° and higher tensile stresses. The degradation of model performance (R^2^ decreasing from >98% to >90%) at γ ≈ 68° under high tensile stress is not due to experimental error but reveals genuine boundaries of the two-domain approximation. This selective degradation occurs where multiple anisotropy mechanisms—rolling-induced uniaxial, stress-induced, and six-fold terms—compete most intensely. During this orientation, under high stress, the actual domain structure is likely to become more complex, and more sophisticated domain configurations may be required. Moreover, in directions apparently different from 0° and 90°, inhomogeneity of mechanical stress may occur. Considering the accuracy of measurements, high values of the *R*^2^ determination coefficient clearly indicate that the 2D variation in the relative magnetic permeability of grain-oriented M120-27s electrical steel can be explained by the proposed model. For FEM simulations of transformer cores, permeability errors below 5% are generally considered acceptable. Figure 4 confirms that model predictions closely track experimental values across all conditions, with R^2^ exceeding 98%, indicating suitability for practical FEM applications.

The directional permeability $\mu_{r}\left(\varphi \right)$ and optimized magnetization angle $\psi_{opt}\left(\varphi \right)$ provided by the model enable complete characterization of the vector B-H relationship in the low-field regime. For an applied magnetic field H at angle φ, the resulting flux density has magnitude $B=\mu_{0}\mu_{r}\left(\varphi \right)H$ and direction $\psi_{opt}\left(\varphi \right)$, where the angular deviation $\psi_{opt}\left(\varphi \right)-\varphi$ quantifies the magnetic anisotropy effect. These outputs allow construction of the 2D permeability tensor required for finite element simulations of transformer cores operating in the quasi-linear regime.

Possible negative values of *p*_0_ and *q*_0_ do not make the confinement coefficient negative due to the dominating the positive value of the *p_iso_* parameter, preserving the clear physical correctness of the developed model.

## 5. Conclusions

The model presented in this paper provides a quantitative description of the mechanical stress dependence on magnetic energy minimization of the 2D relative magnetic permeability in grain-oriented electrical steel. In the presented approach, a planar two-domain system with a movable 180° wall allows for an in-plane magnetization angle ψ to differ from the field angle *φ* and aggregates isotropic, rolling-induced uniaxial, and six-fold confinement terms together with stress-induced anisotropy connected to the axis γ. After transforming to a dimensionless energy, derivative-free parameter identification was carried out during the efficient tri-division search of the optimal values of $\xi_{opt}$ $\psi_{opt}$ in the (ξ,ψ) space.

Validation of the proposed model was carried out on the samples made of M120-27s electrical steel cut at a direction γ to the equal rolling direction: 0°, 11°, 23°, 34°, 45°, 54°, 56°, 68°, and 90°. Validation was made for the applied mechanical tensile stresses σ up to 94.3 MPa using a yoke with a fluxmeter under a magnetizing field amplitude equal to 750 A/m. The identified parameters, *p_iso_*(σ,γ), *p*_0_(σ,γ), *q*_0_(σ,γ), *p_s_*(σ,γ), and *q_s_*(σ,γ), enable very good reproduction of measured 2D relative magnetic permeability. The determination coefficient R^2^ generally exceeds 98%, with a moderate degradation observed for γ equal to 68° at higher values of tensile stresses σ. This selective degradation occurs for the value of γ where multiple anisotropy mechanisms including rolling-induced uniaxial, stress-induced, and six-fold crystallographic terms compete most intensely. It should be highlighted that deterioration of the model for γ equal to 68° validates its physical correctness by revealing genuine boundaries of the two-domain approximation rather than masking limitations through excessive parameterization. The deterioration occurs precisely in the transition region, far from both 0° and 90° orientations where simpler behaviours dominate. This confirms that the model accurately captures the dominant magnetoelastic physics while honestly identifying the specific regime where enhancement through higher-order anisotropy terms or more sophisticated domain structure representation would be beneficial for complete quantitative description.

## Data Availability

The original contributions presented in this study are included in the article. Further inquiries can be directed to the corresponding author.
